# Deciphering peculiar protein-protein interacting modules in *Deinococcus radiodurans*

**DOI:** 10.1186/1745-6150-4-12

**Published:** 2009-04-08

**Authors:** Karim Mezhoud, Haïtham Sghaier, Insaf Barkallah

**Affiliations:** 1 Unit of Microbiology and Molecular Biology, National Center for Nuclear Sciences and Technologies (CNSTN), Sidi Thabet Technopark, 2020, Ariana, Tunisia

## Abstract

Interactomes of proteins under positive selection from ionizing-radiation-resistant bacteria (IRRB) might be a part of the answer to the question as to how IRRB, particularly *Deinococcus radiodurans *R_1 _(Deira), resist ionizing radiation. Here, using the Database of Interacting Proteins (DIP) and the Protein Structural Interactome (PSI)-base server for PSI map, we have predicted novel interactions of orthologs of the 58 proteins under positive selection in Deira and other IRRB, but which are absent in IRSB. Among these, 18 domains and their interactomes have been identified in DNA checkpoint and repair; kinases pathways; energy and nucleotide metabolisms were the important biological processes that were found to be involved. This finding provides new clues to the cellular pathways that can to be important for ionizing-radiation resistance in Deira.

## Reviewers

This article was reviewed by Thiago Motta Venancio (nominated by S. Balaji) and Arcady Mushegian

## Findings

Ionizing radiation-resistant bacteria (IRRB) are "non-spore forming bacteria" that can protect their cytosolic proteins from oxidation and tolerate many DNA double-strand breaks (DSBs) after exposure to high, acute ionizing radiation (doses greater than 1 kilogray (kGy) for a 90% reduction (D10) in the number of Colony Forming Units (CFUs)); moreover, they can resist prolonged desiccation[1]. The dramatic capability to survive extreme conditions is ascribed to their outstanding efficiency in reconstructing functional genomes with high fidelity from hundreds of DSBs generated by DNA-damaging agents [2,3], even while few other organisms can tolerate DSBs [4]. More than fifty years of research has provided many advances on the proteins involved in DNA-repair machinery [5]. However, the mechanism underlying radioresistance is incredible and nevertheless mysterious, in spite of all the studies that have been conducted [4,5].

Throughout the past five decades, *Deinococcus radiodurans *(Deira, D10 ≈ 15 kGy) has been a model for understanding many of the basic principles that govern resistance to ionizing radiation and tolerance of desiccation (for review, refer to [6]). An efficient repair of DNA-strand breaks contributes to the radioresistance of Deira, which harbours DNA-repair pathways that are nearly identical to *Escherichia coli*. However, the interaction among the proteins in their corresponding machineries appears to be different [7]. Moreover, macromolecular complexes and interactions are ubiquitous and are required for the temporal or spatial coordination of cellular functions in all forms of life. Proteins are composed of small units or domains that can physically interact together forming multi-domain protein regions; each region can engage distinct ligands, either simultaneously or at successive stages of signalling [8]. Databases of several complexes and protein-protein interactions were generated to predict the post-genomic perspective of cellular function, in which each biological entity is considered in the context of a complex network of interactions [9-11]. Therefore, we were motivated in the present work to investigate the interactomes of 58 orthologous sets that are present in IRRB such Deira and absent in ionizing-radiation-sensitive bacteria (IRSB) such as *Escherichia coli *and *Thermus thermophilus *[12]. We describe novel interacting proteins and modules in Deira. Indeed, this is the first interactome described for Deira and will provide a framework for the future studies of IRRB.

In this work, we focused on the 58 proteins that are under positive Darwinian selection in IRRB and that are absent in IRSB. To extract these 58 orthologs, we downloaded additional file 1 from a previous work, available at [12]. None of these genes/proteins was previously cited as being implicated in the DNA-preservation or the DNA-repair machineries. 18 of them are of unknown functions (see Additional file 1).

**Figure 1:**
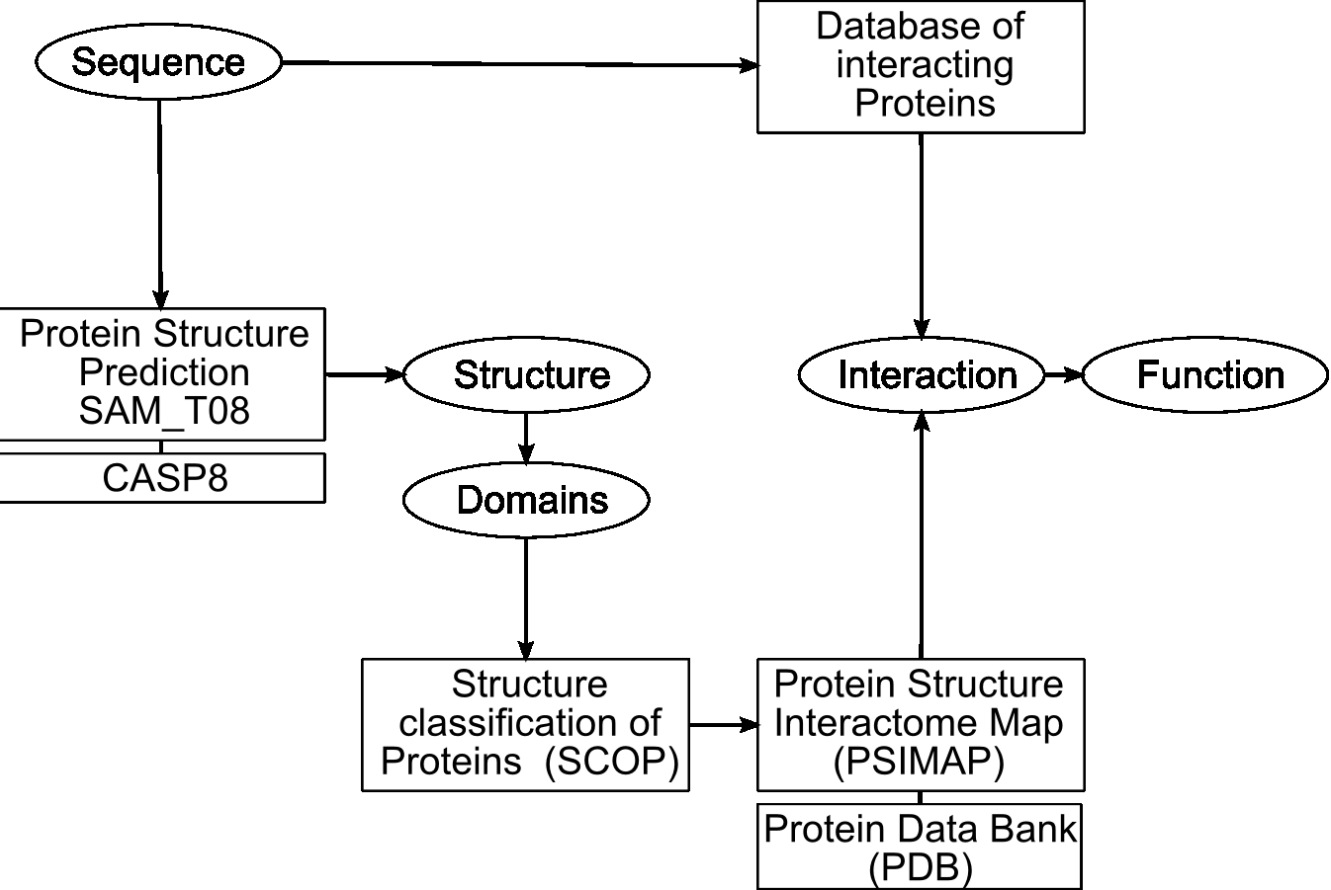
**Diagram of *in silico *analysis done in this work**. Two databases were used to predict interactomes, Database of Interacting Protein (DIP) [10] and Protein Structure Interactome database (PSIbase)[10]. PDB templates and SCOP domains were predicted using the SAM-T08 server [16-19].

Deira ortholog accessions were submitted to the National Centre for Biotechnology Information (NCBI) gene-search engine to load UniProtKB/TrEMBL accessions and corresponding protein sequences. To determine their putative functions, we submitted the sequences to PSI-Blast (Position-Specific Iterated BLAST). The search was based on the non-redundant protein sequence database and the BLOSUM62 Matrix for scoring parameters. To predict the functions and interactomes of the 58 proteins, we initially used their orthologous sequences available at the Database of Interacting Proteins (DIP) [13] (figure 1). The 58 sequence proteins were compared to the dip20080918B.seq file from DIP database. Of these, 44 proteins have a corresponding homolog in the DIP-sequence database (score < e-5). The 44 protein accessions were concatenated in a batch mode using Linux/UNIX shell scripts and C or C++ programs, with the corresponding DIP accessions, and then matched with the latest interaction databases of DIP (dipall20081009.tab.; 78912 interactions). The interactions were then imported using the cytoscape program [14]. The protein interaction maps were generated from a specified set of binary interactions obtained from experimental data collected from different species (*Drosophila melanogaster*, *Saccharomyces cerevisiae*). On the basis of the assumption that proteins from orthologous genes (COGs) have similar interactions or functions if they have a close similarity in their sequences [15], we searched the Deira proteins that contained similarities in the protein-protein interaction databases.

Subsequently, the sequences were submitted to the SAM_T08 server [16-19] for predicting protein structure using Hidden Markov Models [16] and to load the corresponding SCOP domain references [20]. The SCOP domains are used to predict the domain interaction using the PSI Map (PSIbase) (figure 1) [10]. Proteins were then grouped according to the biological processes in which they operate; these details are available in Additional file 2.

Finally, we limited the interactome prediction to the SCOP domains using the Protein Structural Interactome (PSI)-Base data [10].

### Interactome prediction of the orthologs of the 58 proteins present in IRRB and absent in all IRSB using DIP database

On the basis of the assumption that proteins from orthologous genes (Clusters of Orthologous Groups of proteins or COGs) have similar interactions or functions if they have a close similarity in their sequences [15], the interaction maps of the 58 proteins were generated from a specific set of binary interactions obtained from experimental data collected from different species (*Drosophila melanogaster*, *Saccharomyces cerevisiae*) and available on several databases (DIP and PSIbase data).

The 58 protein sequences were compared to the dip20080918B.seq database (42562 sequences) from DIP using the Blast2 program. Among these, 44 proteins were found with their corresponding ortholog in the DIP-sequence database (see Additional file 3). The 44 protein accessions were concatenated with their corresponding DIP accessions and matched with the interaction databases of DIP (dipall20081009.tab). Among these, 9 proteins were found with 1 or more interactions (edges) (see Additional file 4 and Additional file 5). In total, 34 interactions (edges) were found in the DIP database (see Additional file 4). They are related to DNA checkpoint and DNA repair (5 edges), nucleotide metabolism (19 edges), amino acid metabolism and signal transduction (10 edges).

For example, the network involved in DNA checkpoint and repair includes DR_A0338 (kynureninase) that catalyses the reaction of L-Alanine degradation. This reaction generates the purine (NAD and NADP) involved in energy metabolism; or the radial spoke protein 2 (RSP2) (ligase) that has the function of conjugating proteins or DNA. RSPs have a direct link with RNA helicase DHH1 that is involved in recovery at the G1/S DNA-damage checkpoint. DR_A0338 is also involved in mitotic synthesis of damaged DNA through the enzyme CDC7 kinase. Kynureninase provides NAD for protein-modification processes involved in cell division and DNA-damage checkpoint. Other proteins with related pathways are involved in DNA stability and management were found. Thioredoxin, linked to DR_1271, has a direct interaction with DNA polymerase. It is implicated in the neutralisation of reactive oxygen species and in DNA polymerisation. DR_2074 functions to excise 3-methyladenine and 7-methylguanine from the damaged DNA polymer formed by alkylation lesions. It has a direct interaction with the DNA-repairing RAD23 superfamily of proteins that plays a central role both in proteosomal degradation of misfolded proteins and DNA repair. It is involved in DNA-excision repair by stabilising the xeroderma pigmentosum group C protein, thereby perhaps playing a role in DNA-damage recognition and/or in altering chromatin structure to allow access by damage-processing enzymes.

For instance, another network is related to nucleotide metabolism and it includes two proteins, having 19 interactions. DR_1160 participates in purine metabolism, similar to DR_A0338. It has an oxidoreductase function, catalysing the oxidation of uric acid to 5-hydroxyisourate. DR_0505 has a d.114.1.1 domain that is implicated in nucleotide metabolism and transport. The second domain (d.159.1.2) belongs to the superfamily of metallodependent phosphatases and the family of DNA DSB-repair nucleases. Other networks are composed of proteins implicated in signal transduction and amino acid metabolism.

### Deciphering important protein-interacting modules in Deinococcus radiodurans using SCOP domains and PSIbase

Protein domains are regions, typically comprising 40–100 amino acids, within a protein molecule that show structural homology [21]. A domain is the smallest unit of evolution; a large protein can be split into smaller domains. Domains can occur by themselves or in combination with other domains. A superfamily groups together domains of different families with a common evolutionary ancestor, based on structural, functional and evolutionary data.

In the first step, the 58 protein sequences from Deira were submitted to the SAM_T08 server [16-19] for predicting the protein structure using Hidden Markov Models [16] and for loading the corresponding SCOP domain references [20]. Of these, 99 SCOP domains were returned and 88 interactions were found between these domains using psimap1.71 databases (Additional file 2, Additional file 6). There are 54 interprotein interactions and 34 intraprotein interactions.

All domain-domain interactions are considered by the interface that belongs to the same protein and also to different proteins. Initially, the domains were grouped by the protein classes they belong to. They were subsequently matched using shell script with the psibase 1.71 data to find putative domain interactions. Thus, 9 networks implicated in different biological processes were found.

Domain networks were then grouped by biological processes and metabolism pathways. Three of them are involved in the recognition of DNA and its binding with related proteins, in addition to the catabolism of ATP (group B, Additional file 6).

The P-loop containing the nucleotide triphosphate hydrolase (c.37.1.20 and c.37.1.12 group B) is linked with the "Winged-helix" DNA-binding domain (a.4.5), DNA-repair protein MutS (a.113.1.2 and c.55.6.1), Cdc-48 domain (d.31.1.1), DNA polymerase (a.80.1.1 and d.131.1.2) N-terminal nucleophile aminohydrolases (NTN hydrolases, d.153.1), and with ABC transport (f.22.1.1). These superfamilies are involved in a restricted range of critical DNA management.

We found two network interactions of two proteins, which are common between the DIP and PSIbase databases (DR_A0178 and DR_A0129). Research results show that, in both cases, DR_A0178 belongs to a network of interactions involved in electron transport (biological process) and redox activity (molecular-based); and that DR_A0129 belongs to a network of interactions involved in metabolic process (biological process) and cationic transmembrane transporter activity. These results show the correlation of data between these two databases (DIP and PSIbase).

The present discovery notes deals with use of combinatorial server for structural protein prediction and protein-protein interaction databases to deciphering the news clues about the radioresistance bacteria. Based of protein sequence from Deira, we predicted several cluster interacting proteins present on radioresistant bacteria but absent on radiosensible bacteria. This method allowed us to draw the pathways that maybe have a link with radioresistance machinery. Indeed we have predicted four proteins that have a direct involvement with the DNA repair (DR_0511, DR_0918, DR_0268 and DR_2074). Two of them (DR_0511, DR_0918) have c.37.1 domains that belong to the ABC transporter ATPase domain-like family that can be found in the RecA protein (information was found on pre-SCOP web site: ), widely studied and has an important role in the radioresistance in Deira [22]. These proteins are the new interesting candidates for future experimental work.

## Competing interests

The authors declare that they have no competing interests.

## Authors' contributions

KM carried out the conception and design of the methods, acquisition and analysis of data. HM has been involved in data analysis, drafting the manuscript and revising it critically for important intellectual content. IB participated in coordination and helped to draft the manuscript.

## Reviewers' comments

### Reviewer 1

Dr. Thiago Motta Venancio (nominated by S. Balaji), NCBI-NIH, Bethesda, Maryland, United States

In my first revision of this manuscript, I pointed several key problems with this study (see below). In addition to the problems emphasized in the first report, one of the most fundamental problems is that the study is almost completely based on the electronic transfer from eukaryotes to bacterium. The efficacy of such process was evaluated and shown to be reliable only for extremely high levels of sequence similarity [23,24], which does not seem to be the case of the proteins analyzed in this paper.

The text still requires English revision. In addition, there are several typographic errors that could have been solved by a spell-checker.

Although the revised version of the manuscript is slightly better than the previous one, I think it is still below the journal's publication standards. Considering the issues outlined in my revisions, I do not support the publication of this manuscript.

Response:

Luscombe et al. indicated on his work that: Using interaction information from Saccharomyces cerevisiae,

Caenorhabditis elegans, Drosophila melanogaster, and Helicobacter pylori, we find that protein-protein interactions can be transferred when a pair of proteins has a joint sequence identity >80% or a joint

E-value <10(-70).

We compared Deinococccus radiodurans protein sequence to the protein sequences from DIP (Drosophila melanogaster, Saccharomyces cerevisiae) using Blast2 program [25]. We selected only the sequence that had E-value < e-5. We add in additional file (blast2 file)only the sequence that selected of the output of the sequence comparison [26].

In the case of PSIMAP we used directly the SCOP domain references.

**Reviewer: **In this manuscript, Mezhoud and colleagues aimed to identify protein-protein interactions that would be related to the ionizing radiation resistance in the bacteria Deinococcus radiodurans. The scientific problem and the way it was addressed in this study are not clearly stated. The authors should provide a minimal description about how the study has been conducted, rather than just citing other publications and databases.

**Response: **The 58 orthologs sets were selected using The positive Darwinian philosophy that leads to fix the advantageous mutations and the fundamental process behind adaptive changes in genes [27]. The 58 orthologs sets were selected using DnaSP program . This program calculates the ratios of no synonymous to synonymous mutation rates (Ka/Ks) in protein coding genes. The Ka/Ks ratio measures the strength of selection [28].

**Reviewer: **The authors mentioned that the proteins are under positive selection and not present in IRSB bacteria. However, the results were basically obtained using orthology assessment and domain detection. Since these proteins are absent in most bacteria, what are the model organisms used to infer the interactome?

**Response: **Radioresistance is surprisingly high in many organisms, in contrast to previously held views. For example, the study of environment, animals and plants around the Chernobyl accident area has revealed an unexpected survival of many species, despite the high radiation levels. A Brazilian study in a hill in the state of Minas Gerais which has high natural radiation levels from uranium deposits, has also shown many radioresistant insects, worms and plants. We cite the examples of *Drosophyla *and *Sacharomyces*.

*Drosophyla*:  Radioresistance of a natural population of Drosophila willistoni living in a radioactive environment. Mutat Res. 1973 Sep;19(3):325–9.

*Sccharomyces*: 

**Reviewer: **How complete are the interactomes in these model organisms? How complete is the database used?

**Response: **We explained on the following, the strategy given by every server to complete their database.

**Searching the Database of interacting proteins on DIP: **Currently protein-protein interactions are entered into the DIP only following publication in peer-reviewed journals. Entry is done manually by the curator, followed by automated tests that show the proteins and citations exist. Interactions are double-checked by a second curator and flagged accordingly in the database. DIP can be searched in a variety of ways. One can look for interactions involving a specific protein by entering its gene name or its accession code from GenBank, PIR or SWISS-PROT. More general searches can be performed for information such as organisms, protein superfamilies, keywords, experimental techniques or literature citations. Multiple fields can be searched simultaneously to narrow the query, and the use of wildcards and regular expressions is supported to further aid in searching. A search returns a list of protein-protein interactions, each hyperlinked to a DIP entry. Each resulting DIP entry reports information about the two interacting proteins, the protein domains and range of amino acids involved the curator, date of entry and updating and the articles describing the interaction, and the corresponding experiments. For example, a search on a single protein returns all of the interactions recorded in DIP in which that protein participates [29].

**Searching the Database of interacting proteins on PSIBASE: **PSIBASE, the Protein Structural Interactome Map, is a database of all the structurally observed interactions between protein domains of known three-dimensional structure in the PDB. It can be constructed using any reliable protein domain definition, where domains are defined as evolutionarily conserved structural and functional protein units. Here we use the domain definitions provided by SCOP (Structural Classification of Proteins), which uses structural and functional homology to manually define evolutionarily distinct protein domain families and superfamilies. Alternatively, other domain definitions (such as CATH, FSSP, Pfam, etc.) can be used [30].

**Reviewer: **What means a "correct score in the DIP-sequence database"?

**Response: **We will change this vocabulary. We compared le 58 protein sequences to the DIP protein sequence database using Blast2 program. We found 44 sequences with a e-value lower than e-5.

**Reviewer: **All these questions should be discussed, along with the limitations involving the use of computationally predicted protein-protein interactions. This is particularly critical when analyzing proteins that may form completely different complexes due to their putative relationship with the IRRRB phenotype.

**Response: **Entry is done manually by the curator, followed by automated tests that show the proteins and citations exist. Interactions are double-checked by a second curator and flagged accordingly in the database (see the paragraphs "Searching the databases").

**Reviewer: **The fact that these 58 proteins are under positive selection may provide clues about the differences between IRRB and IRSB. However, it is still premature to state that "... these orthologous sets decide the difference between IRRB and IRSB", as authors did in the background section.

**Response: **OK we will change this sentence. Absolutely we are not sure that these ortholog sets make difference between IRRB and IRSB. This is only a prediction, based on a program to calculate "recognized" to reduce the workload of a full proteome of Deira to 58 proteins and database interactome reasonably established. There is still checking the genes of interest by an experimental study, which needs enough time consumed.

**Reviewer: **The results could be better tied up. I had the general impression that protein annotations are just placed in different phrases, without a biological discussion on their possible roles in an orchestrated manner with their interacting partners. This is of key importance when discussing proteins under a systems perspective.

**Response: **We restricted the discussion to some proteins or domains involved on DNA repair and not cited previously. This work is a discovery note using available protein interaction data. We don't have enough information to discuss the possible roles in an orchestrated manner. Nonetheless, we made changes on our manuscript. Our goal was to draw the clusters protein-protein interactions to locate the most interesting proteins which involved on DNA repair.

**Reviewer: **Moreover, the conclusions section is poor in the present format and does not present any clearly novel insight in the differences between IRRB and IRSB. To exemplify my point, I took the phrase "Considering all the molecular processes that contribute to the radioresistant phenotype of radioresistant phenotype of Deira, it can be concluded that it is achieved by the interaction of various proteins.". This is an obvious outcome, since (almost) every process regulated by proteins is achieved by interaction of various proteins.

**Response: **The conclusion has been rewritten. We worked on proteins that exist only in IRRB. If there is an interest protein or domain that involved on DNA repair or the neutralization of reactive oxygen species then IRSB do not have.

**Reviewer: **The results and conclusions sections of the abstract are also not very clear. I suggest some re-phrasing to better explain the analyses and their respective implications. Instead of mentioning very broad terms, such as "pathways involving kinases", some specific result should be briefly explained.

**Response: **The abstract has been rewritten.

**Reviewer: **For the reasons outlined here, I think the manuscript requires extensive modifications to meet the publication standards of Biology Direct. English revision is also strongly recommended in several parts of the text.

Minor changes

**Reviewer: **I think citations should not be used in the abstract.

**Response: **OK

**Reviewer: **In the Additional file 1, I suggest to add separate columns for database identifiers, instead of pasting the FASTA formatted sequence in spreadsheet comments. It makes easier for one to process the file and retrieve the sequence from public databases.

**Response: **OK We added a column of the UniProtKB/TrEMBL accession.

**Reviewer: **There is a problem in the design of the flowchart (figure 1). The two arrows in the top box are referring to the same process (blastp).

**Response: **OK

**Reviewer: **Some terms are mis-used in the text and figures. For example, E-values are lower or greater than a threshold, not poorer (Additional file 2).

**Response: **OK we delete the term "poor"

**Reviewer: **Although used in speech, the word "blast" should not be used as a verb in scientific publications when referring to sequence alignments.

**Response: **OK we changed the phrases.

### Reviewer 2

Dr Arcady Mushegian, Stowers Institute for Medical Research, Kansas City, United States

Reviewer:

I think this is a Discovery Note, not a full paper, its length notwithstanding.

Response:

We changed the sections as a Discovery note.

Reviewer:

"Results" section of the "Abstract": "proteic" must be a typo.

Response:

OK we changed this word.

Reviewer:

p. 4. "One of the basic characteristics of biological organisation is that everything in an organism can be regarded as a part of a complex network [8,9]." – I am not sure what this means or whether this even is true (why 'everything' and not 'some things'? why Basic characteristic and not a conjecture?). Also, what do references 8 and 9 have to do with establishing this?

Response:

OK, We deleted this phrase.

Reviewer:

p. 5 "Proteins that participate in more interactions are phenotypically (ionising-radiation resistance, in this case) more important [20] and evolutionarily more conserved [21,22]" – be careful with these generalizations, trends there are subtle. See, for example, Hurst LD, Smith NG. Do essential genes evolve slowly? Curr. Biol. (1999) 9:747–750 and Hirsh AE, Fraser HB. Protein dispensability and rate of evolution. Nature (2001) 411:1046–1049. In any case, is this correlation even needed for the authors' argument?

Response:

OK

Reviewer:

p. 6 top line and several other places. Avoid the slang – replace "blasted against" with "compared to". Ibid, line 3: what is "correct score"?

Response:

OK

Reviewer:

p. 6 par. 2 and further. Essentially, there was no explicit assessment of the reliability of predicted interaction, i.e., all interactions recorded in DIP or PSIMAP, whether observed in any species or inferred, were treated as true, correct? Moreover, in the case of DIP, the interaction information was transferred from the orthologs in other species, but in the case of fold recognition and PSIMAP, also from paralogs?

Response:

Searching the Database of interacting proteins on DIP:

Currently protein-protein interactions are entered into the DIP only following publication in peer-reviewed journals. Entry is done manually by the curator, followed by automated tests that show the proteins and citations exist. Interactions are double-checked by a second curator and flagged accordingly in the database.

DIP can be searched in a variety of ways. One can look for interactions involving a specific protein by entering its gene name or its accession code from GenBank, PIR or SWISS-PROT. More general searches can be performed for information such as organisms, protein superfamilies, keywords, experimental techniques or literature citations. Multiple fields can be searched simultaneously to narrow the query, and the use of wildcards and regular expressions is supported to further aid in searching. A search returns a list of protein-protein interactions, each hyperlinked to a DIP entry. Each resulting DIP entry reports information about the two interacting proteins, the protein domains and range of amino acids involved, the curator, date of entry and updating and the articles describing the interaction, and the corresponding experiments. For example, a search on a single protein returns all of the interactions recorded in DIP in which that protein participates [31].

Searching the Database of interacting proteins on PSIBASE:

PSIBASE, the Protein Structural Interactome Map, is a database of all the structurally observed interactions between protein domains of known three-dimensional structure in the PDB. It can be constructed using any reliable protein domain definition, where domains are defined as evolutionarily conserved structural and functional protein units. Here we use the domain definitions provided by SCOP (Structural Classification of Proteins), which uses structural and functional homology to manually define evolutionarily distinct protein domain families and superfamilies. Alternatively, other domain definitions (such as CATH, FSSP, Pfam, etc.) can be used [32].

PSIMAP ALGORITHM

The basic mechanism to check interactions between any two domains or proteins is the calculation of the Euclidean distance in order to see if they are within a certain distance threshold. PSIMAP checks every possible pair of structural domains in a protein to see if there are at least five residue contacts within a 5 Å distance (5-5 rule). The current PSIMAP protocol has three methods. They are the Full Atom Contact (FAC) PSIMAP, Sampled Atom Contact (SAC) PSIMAP and Bounding Box Contact (BBC) PSIMAP [33].

Reviewer:

p. 6–7: there is some biological rationalization of the observed interactions (calling them "networks" is a bit gratuitous; they are clusters of interacting proteins, but the network structure of these clusters is never explored), but, despite the section title, I do not see any comparison to the IRSB and how they get by without these clusters.

Response:

We replaced "networks" by "clusters of interacting proteins"

We are not sure that these ortholog sets make difference between IRRB and IRSB. This is only a prediction, based on a program to calculate "recognized" to reduce the workload of a full proteome of Deira to 58 proteins and database interactome reasonably established. There is still checking the genes of interest by an experimental study, which needs enough time consumed.

We worked on proteins that exist only in IRRB. If there is an interest protein or domain that involved on DNA repair or the neutralization of reactive oxygen species then IRSB do not have.

## Supplementary Material

Additional File 1 **The list of the 58 Orthologs.** Click here for file

Additional File 2 **SAM T_08 blast result: Best-scoring hits from combining t06 t04 t2k version.** If the E-value is poor (greater than 1.0e^-02^, for example), then the model should be regarded as speculative [15-18]. Click here for file

Additional File 3 **Blast result of 58 *Deinococcus radiodurans *proteins with the DIP database (dipall20081009.tab.; 78912 interactions).** Click here for file

Additional File 4 **Interactomes of 9 proteins from the DIP database**. Each protein is represented by a square (node), according to its identity in the DIP database. The 9 proteins of interest are represented by a bigger square. Interaction is represented by Edge. Protein-protein interaction data were extracted from the DIP database. Networks were grouped by biological processes. Groups were implicated respectively in signal transduction, DNA checkpoint in G1/S, DNA repair and nucleotide metabolism. Click here for file

Additional File 5 **Interactomes of the 9 proteins using the DIP database.** Click here for file

Additional File 6 **Interactomes of the 58 proteins under positive selection in ionizing radiation-resistant bacteria (IRRB) but absent in all ionizing radiation-sensitive bacteria (IRSB)**. SCOP domains were represented by octagons. Union of octagons represent proteins with multiple domains. Protein-protein interactions were represented by edges. Data on interaction domains were extracted from the PSIBASE 1.71 database. Network interactions were grouped by biological processes. Groups were involved respectively in electron transport and oxidoreduction (A), DNA repair (B), Energy metabolism (C) and beta-lactam resistance (D). Click here for file
