# Supplementary material for: Deciphering peculiar protein-protein interacting modules in Deinococcus radiodurans
Source: Biol Direct. 2009 Apr 8;4:12. doi: 10.1186/1745-6150-4-12 (PMC2672081; doi:10.1186/1745-6150-4-12)
Supplement: Additional File 3 — Blast result of 58 Deinococcus radiodurans proteins with the DIP database (dipall20081009.tab.; 78912 interactions). [file 1745-6150-4-12-S3.doc]

**Additional file 3:** Blast result of 58 *Deinococus radiodurans* protein to DIP database ( dipall20081009.tab.; 78912 interactions) :

| **Dra protein**  **gene - uniprot** | **Homologous in DIP database** | **Score** | **E value** | **Interaction** | **target** |
| --- | --- | --- | --- | --- | --- |
| DR_A0338-Q9RYH5 | DIP-6518N|refseq:NP_013332|uniprot:Q05979 | 114 | 3e-25 | DIP-11720E  DIP-63815E | DIP-1235N P06243 NP_010267  DIP-2238N P39940 NP_011051 |
| DR_A0178-Q9RYX6 | DIP-25285N|refseq:NP_502747|uniprot:O17892 | 59 | e^-129^ | DIP-81944E | DIP-26974N Q9N3T2 NP_491168 |
| DR_A0147-Q9RZ06 | DIP-27218N|refseq:NP_509820|uniprot:Q20502 | 353 | 3e-97 | DIP-43468E  DIP-81773E | DIP-26375N Q9TZ39 NP_497188  DIP-26227N P39745 |
| DR_2074-Q9RSQ0 | DIP-40787N|refseq:NP_002425|uniprot:P29372 | 84 | 7e-17 | DIP-69118E  DIP-69119E | DIP-39944N P54727 NP_002865  DIP-34442N P54725 NP_005044 |
| DR_1666-Q9RTU0 | DIP-44440N|uniprot:O83068 | 76 | 4e-14 | DIP-75369E | DIP-44439N O83927 |
| DR_1271-Q9RUW0 | DIP-31841N|uniprot:P0ACY1 | 42 | 7e-04 | DIP-82592E | DIP-31856N P0AA25 |
| DR_1160-Q9RV70 | DIP-20028N|refseq:NP_476779|uniprot:P16163 | 132 | 5e-31 | DIP-23293E  DIP-25793E  DIP-28809E  DIP-30593E  DIP-31828E  DIP-34291E  DIP-34848E  DIP-34860E  DIP-37268E  DIP-37366E  DIP-37403E  DIP-39074E  DIP-39075E | DIP-18563N NP_610120  DIP-19975N NP_648859  DIP-17101N NP_726771  DIP-17929N NP_725104  DIP-18312N NP_652510  DIP-23240N NP_476894  DIP-17273N NP_723746  DIP-20681N NP_724173  DIP-23774N NP_476572  DIP-17808N NP_609339  DIP-23167N NP_731675  DIP-17468N NP_611470  DIP-23289N NP_650297 |
| DR_0551-Q9RWW4 | DIP-25054N|refseq:NP_496469|uniprot:Q20772 | 199 | 4e-51 | DIP-40421E  DIP-40675E  DIP-41738E  DIP-43261E  DIP-44290E | DIP-26892N Q17446 NP_501365  DIP-25964N Q17868 NP_501457  DIP-26227N P39745  DIP-26532N Q9XXA2 NP_496438  DIP-27408N P34659 NP_498708 |
| DR_0505-Q9RX10 | DIP-18548N|refseq:NP_724960 | 105 | 2e-22 | DIP-20607E  DIP-23833E  DIP-23834E  DIP-23835E  DIP-23836E | DIP-17588N NP_47677  DIP-17346N NP_569838  DIP-20237N NP_572777  DIP-17429N P17886 NP_477118  DIP-17580N NP_648567 |
| DR_A0363-Q9RYF5 | DIP-14862N|refseq:NP_215948 | 369 | e^-102^ | 0 |  |
| DR_A0361-Q9RYF7 | DIP-14663N|refseq:NP_215755 | 87 | 4e-17 | 0 |  |
| DR_A0326-Q9RYI6 | DIP-14482N|refseq:NP_215888 | 127 | 3e-29 | 0 |  |
| DR_A0308-Q9RYK4 | DIP-16897N|refseq:NP_215535 | 70 | 2e-12 | 0 |  |
| DR_A0222-Q9RYT6 | DIP-14592N|refseq:NP_218151 | 57 | 2e-08 | 0 |  |
| DR_A0216-Q9RYU2 | DIP-12021N|uniprot:P77619 | 74 | 2e-13 | 0 |  |
| DR_A0148-Q9RZ05 | DIP-9855N|refseq:NP_417359|uniprot:P76641 | 44 | 4e-04 | 0 |  |
| DR_A0129-Q9RZ24 | DIP-12851N|refseq:NP_417351|uniprot:Q46808 | 84 | 2e-16 | 0 |  |
| DR_A0121-Q9RZ32 | DIP-14493N|refseq:NP_217846 | 197 | 3e-50 | 0 |  |
| DR_A0067-Q9RZ87 | DIP-13513N|refseq:NP_217019|uniprot:O06166 | 270 | 6e-73 | 0 |  |
| DR_A0007-Q9RZE2 | DIP-15951N|refseq:NP_215974 | 162 | 4e-40 | 0 |  |
| DR_2612-Q9RR84 | DIP-14089N|refseq:NP_217153|uniprot:P71936 | 57 | 1e-08 | 0 |  |
| DR_2553-Q9RRD9 | DIP-14495N|refseq:NP_216952 | 54 | 3e-07 | 0 |  |
| DR_2538-Q9RRF4 | DIP-13910N|refseq:NP_216396|uniprot:P63721 | 177 | 3e-44 | 0 |  |
| DR_2531-Q9RRG1 | DIP-13567N|refseq:NP_216807|uniprot:Q59570 | 167 | 1e-41 | 0 |  |
| DR_2261-Q9RS66 | DIP-11291N|uniprot:P77735 | 155 | 6e-38 | 0 |  |
| DR_2242-Q9RS85 | DIP-36924N|refseq:NP_058865|uniprot:P35704 | 99 | 1e-21 | 0 |  |
| DR_2189-Q9RSD5 | DIP-15529N|refseq:NP_214948 | 56 | 3e-08 | 0 |  |
| DR_2091-Q9RSN3 | DIP-14484N|refseq:NP_216181 | 171 | 9e-43 | 0 |  |
| DR_1784-Q9RTI0 | DIP-7649N|refseq:NP_010520|uniprot:P49367 | 89 | 3e-18 | 0 |  |
| DR_1765-Q9RTJ7 | DIP-14864N|refseq:NP_217844 | 52 | 4e-07 | 0 |  |
| DR_1700-Q9RTQ6 | DIP-14352N|refseq:NP_214582 | 93 | 6e-19 | 0 |  |
| DR_1096-Q9RVD3 | DIP-9149N|refseq:NP_286783|uniprot:P07638 | 145 | 9e-35 | 0 |  |
| DR_1028-Q9RVK0 | DIP-36045N|uniprot:P0AFT2 | 160 | 8e-40 | 0 |  |
| DR_0918-Q9RVV4 | DIP-14727N|refseq:NP_217680 | 65 | 5e-71 | 0 |  |
| DR_0901-Q9RVX0 | DIP-10638N|refseq:NP_289743|uniprot:P09170 | 41 | 4e-04 | 0 |  |
| DR_0823-Q9RW46 | DIP-13384N|refseq:NP_217218|uniprot:Q59568 | 248 | 7e-66 | 0 |  |
| DR_0565-Q9RWV0 | DIP-36045N|uniprot:P0AFT2 | 97 | 1e-50 | 0 |  |
| DR_0511-Q9RX04 | DIP-10964N|refseq:NP_414900|uniprot:Q47538 | 105 | 2e-22 | 0 |  |
| R_0464-Q9RX51 | DIP-13875N|refseq:NP_216078|uniprot:Q10769 | 282 | 9e-76 | 0 |  |
| DR_0463-Q9RX52 | DIP-13876N|refseq:NP_216079|uniprot:Q10768 | 305 | 2e-82 | 0 |  |
| DR_0433-Q9RX82 | IP-13021N|refseq:NP_216584|uniprot:Q10670 | 48 | 1e-05 | 0 |  |
| DR_0192-Q9RXW3 | DIP-16226N|refseq:NP_216109 | 77 | 2e-14 | 0 |  |
| DR_0108-Q9RY43 | DIP-13298N|refseq:NP_214716|uniprot:P65374 | 130 | 6e-30 | 0 |  |
| DR_1355-Q9RUM9 | DIP-16041N|refseq:NP_214822 | 47 | 2e-05 | 0 |  |
| DR_A0368-Q9RYF0 | DIP-6869N|refseq:NP_418417|uniprot:P30140 | 30 | 7.1 |  |  |
| DR_A0151-Q9RZ02 | DIP-40615N|uniprot:Q9XEK5 | 33 | 0.92 |  |  |
| DR_A0008-Q9RZE1 | DIP-20440N|refseq:NP_722605 | 32 | 0.97 |  |  |
| DR_A0004-Q9RZE5 | DIP-15596N|refseq:NP_214885 | 32 | 0.40 |  |  |
| DR_2624-Q9RR73 | DIP-41177N|uniprot:Q9Y5V3 | 31 | 0.94 |  |  |
| DR_2265-Q9RS62 | DIP-16948N|refseq:NP_215444|uniprot:O86344 | 32 | 2.8 |  |  |
| DR_1597-Q9RU00 | DIP-15476N|refseq:NP_215962 | 34 | 0.19 |  |  |
| DR_1158-Q9RV72 | DIP-35755N|refseq:NP_001082370|uniprot:Q8AWF4 | 30 | 1.6 |  |  |
| DR_0801-Q9RW68 | DIP-14975N|refseq:NP_215654 | 35 | 0.16 |  |  |
| DR_0625-Q9RWP1 | DIP-25227N|refseq:NP_501127|uniprot:Q18566 | 31 | 1.2 |  |  |
| DR_0620-Q9RWP6 | DIP-15815N|refseq:NP_217082 | 42 | 0.005 |  |  |
| DR_0598-Q9RWR8 | DIP-3309N|refseq:NP_208334|uniprot:O26068 | 39 | 0.003 |  |  |
| DR_0268-Q9RXP2 | DIP-8839N|refseq:NP_010037|uniprot:Q07748 | 42 | 0.001 |  |  |
|  |  |  |  |  |  |
