# Supplementary figures and images for: Deciphering peculiar protein-protein interacting modules in Deinococcus radiodurans
Source: Biol Direct. 2009 Apr 8;4:12. doi: 10.1186/1745-6150-4-12 (PMC2672081; doi:10.1186/1745-6150-4-12)

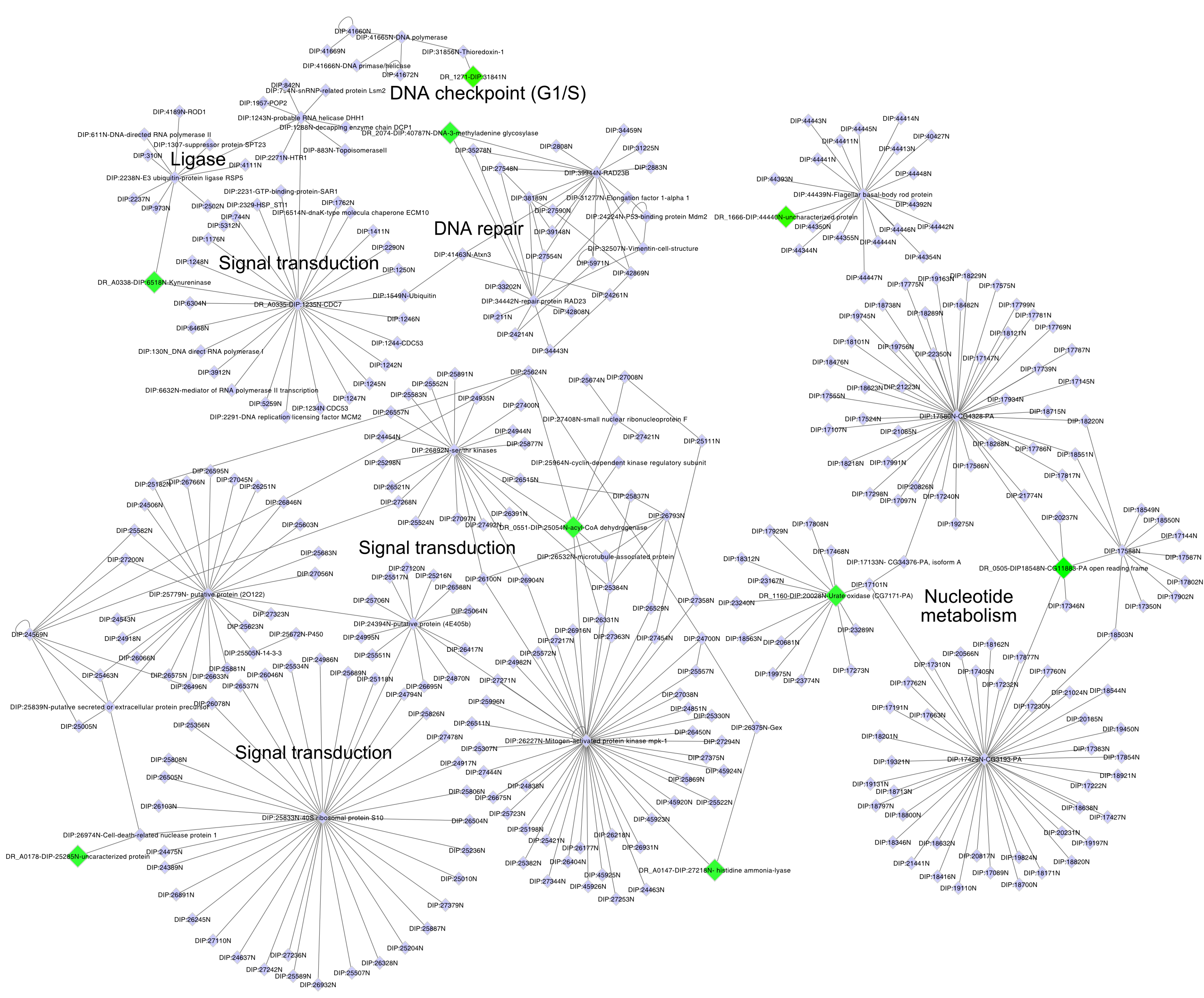

Supplement: Additional File 4 — Interactomes of 9 proteins from the DIP database. Each protein is represented by a square (node), according to its identity in the DIP database. The 9 proteins of interest are represented by a bigger square. Interaction is represented by Edge. Protein-protein interaction data were extracted from the DIP database. Networks were grouped by biological processes. Groups were implicated respectively in signal transduction, DNA checkpoint in G1/S, DNA repair and nucleotide metabolism. [file 1745-6150-4-12-S4.png]

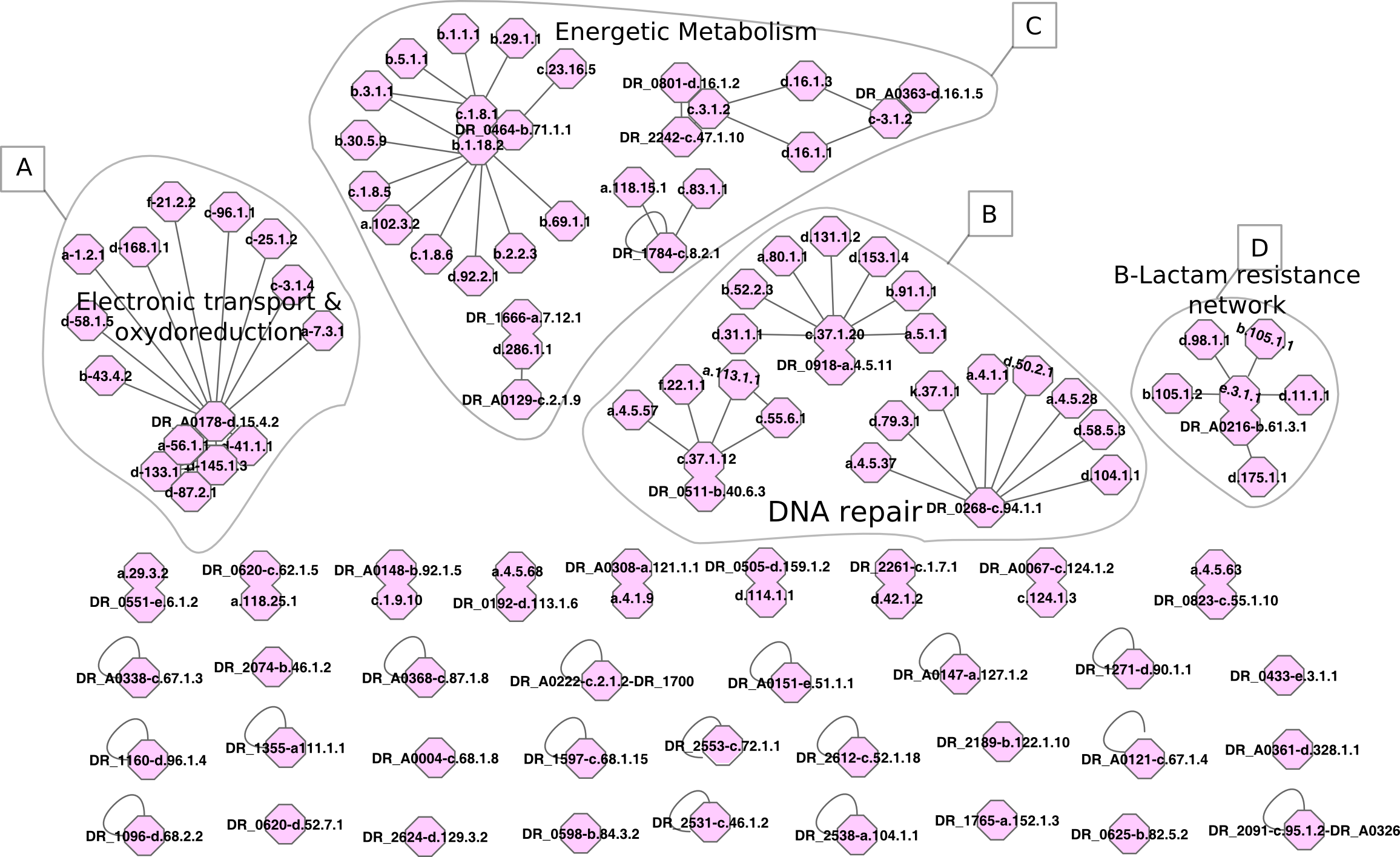

Supplement: Additional File 6 — Interactomes of the 58 proteins under positive selection in ionizing radiation-resistant bacteria (IRRB) but absent in all ionizing radiation-sensitive bacteria (IRSB). SCOP domains were represented by octagons. Union of octagons represent proteins with multiple domains. Protein-protein interactions were represented by edges. Data on interaction domains were extracted from the PSIBASE 1.71 database. Network interactions were grouped by biological processes. Groups were involved respectively in electron transport and oxidoreduction (A), DNA repair (B), Energy metabolism (C) and beta-lactam resistance (D). [file 1745-6150-4-12-S6.png]
