# Supplementary material for: Deciphering peculiar protein-protein interacting modules in Deinococcus radiodurans
Source: Biol Direct. 2009 Apr 8;4:12. doi: 10.1186/1745-6150-4-12 (PMC2672081; doi:10.1186/1745-6150-4-12)
Supplement: Additional File 5 — Interactomes of the 9 proteins using the DIP database. [file 1745-6150-4-12-S5.docx]

Additional file 5: Interactomes of the 9 proteins using DIP database

| Deira references | interaction | Deira target | orthologous target |  |
| --- | --- | --- | --- | --- |
| DR_A0338-Q9RYH5 | DIP-11720E | DR_A0335-NP_296367  serine/threonine protein kinase, putative  1e-05 | P06243 | CDC7 is a  protein kinase needed for the initiation of mitotic DNA synthesis [1] |
|  | DIP-63815E |  | P39940 | ubiquitin-protein ligase RSP5  Interact with HSE1 (P38753, Protein transport) [2]  Interact with BUL1 (P48524, ligase-binding protein) AND BUL2.  [3]Interact with ROD1(Q02805, Mediates resistance to o-dinitrobenzene, calcium and zinc.) AND ROG3 (P43602, Involved in resistance to GST substrate o-dinitrobenzene (o-DNB).).  [4]Interact with RUP1 (Q12242, Modulates the activity of the RSP5 HECT ubiquitin-protein ligase) AND UBP2 (Q01476, Has an ATP-independent isopeptidase activity)[5].  Interact with RCR1 (P38212, Regulates chitin deposition in the cell wall)[6]. |
| DR_A0178-Q9RYX6 | DIP-81944E |  | Q9N3T2 | Cell-death-related nuclease protein 1 |
| DR_A0147-Q9RZ06 | DIP-43468E |  | Q9TZ39 | GEX Interacting protein family member (gei-4) |
|  | DIP-81773E |  | P39745 | Mitogen-activated protein kinase mpk-1  ATP-binding; Cell cycle  CATALYTIC ACTIVITY] ATP + a protein = ADP + a phosphoprotein  [COFACTOR] Magnesium (By similarity)  [ENZYME REGULATION] Activated by tyrosine and threonine  phosphorylation  [INTERACTION] O17109 |
| DR_2074-Q9RSQ0 | DIP-69118E |  | P54727 | excision repair protein RAD23 homolog B  INTERACTION WITH NGLY1 (Q503I8, transglutaminase-like superfamily).  [7] |
|  | DIP-69119E |  | P54725 | excision repair protein RAD23 homolog A  DNA damage; DNA repair  INTERACTION WITH HIV-1 VPR.  [8] |
| DR_1666-Q9RTU0 | DIP-75369E |  | O83927 | Flagellar basal-body rod protein (FlgG-2) |
| DR_1271-Q9RUW0 | DIP-82592E | DR_0944-NP_294668  5e-26 | P0AA25 | Thioredoxin-1 |
| DR_1160-Q9RV70 | DIP-23293E |  | NP_610120 | General receptor for phosphoinositides 1 CG11628-PA |
|  | DIP-25793E |  | NP_648859 | CG13050 CG13050-PA |
|  | DIP-28809E | DR_1891-NP_295614  TPR repeat-containing protein  2e-07 | NP_726771 | CG14815 CG14815-PA |
|  | DIP-30593E |  | NP_725104 | Ornithine decarboxylase antizyme CG16747-PC |
|  | DIP-31828E |  | NP_652510 | CG17777 CG17777-PA |
|  | DIP-34291E |  | NP_476894 | Sox box protein 14 CG3090-PA |
|  | DIP-34848E |  | NP_723746 | virus-induced RNA 1 CG31764-PA |
|  | DIP-34860E |  | NP_724173 | CG31797 CG31797-PA |
|  | DIP-37268E |  | NP_476572 | hermaphrodite CG4694-PA |
|  | DIP-37366E |  | NP_609339 | obstructor-B CG4778-PA |
|  | DIP-37403E |  | NP_731675 | CG4810 CG4810-PA |
|  | DIP-39074E |  | NP_611470 | Cuticular protein 56F CG9036-PA |
|  | DIP-39075E |  | NP_650297 | CG9269 CG9269-PA |
| DR_0551-Q9RWW4 | DIP-40421E | DR_0058-NP_293784  serine/threonine protein kinase, putative  1e-13 | NP_501365 | P38 Map Kinase family member (pmk-1) |
|  | DIP-40675E |  | NP_501457 | Cyclin-dependent protein Kinase (CDC28) regulatory Subunit family member (cks-1) |
|  | DIP-41738E | DR_2518-NP_296238  serine/threonine protein kinase, putative  5e-14 | P39745 | Mitogen-activated protein kinase mpk-1  [CATALYTIC ACTIVITY] ATP + a protein = ADP + a phosphoprotein.  [COFACTOR] Magnesium (By similarity)  [ENZYME REGULATION] Activated by tyrosine and threonine  phosphorylation  [INTERACTION] O17109  belongs to the protein kinase superfamily  [DOMAIN] The TXY motif contains the threonine and tyrosine residues whose phosphorylation activates the MAP kinases. |
|  | DIP-43261E |  | NP_496438 | microtubule End Binding Protein family member (ebp-2) |
|  | DIP-44290E |  | NP_498708 | Small Nuclear Ribonucleoprotein family member (snr-5) |
| DR_0505-Q9RX10 | DIP-20607E |  | NP_47677 | flgG protein product (AA 1-260) |
|  | DIP-23833E |  | NP_569838 | CG12470 CG12470-PA |
|  | DIP-23835E |  | NP_477118 | crooked neck CG3193-PA |
|  | DIP-23836E |  | NP_648567 | CG4328 CG4328-PA |

1. Bahman M, Buck V, White A, Rosamond J: **Characterisation of the CDC7 gene product of Saccharomyces cerevisiae as a protein kinase needed for the initiation of mitotic DNA synthesis**. Biochim Biophys Acta 1988, **951**(2-3):335--343.

2. Kee Y, Lyon N, Huibregtse JM: **The Rsp5 ubiquitin ligase is coupled to and antagonized by the Ubp2 deubiquitinating enzyme**. EMBO J 2005, **24**(13):2414--2424.

3. Wang G, Yang J, Huibregtse JM: **Functional domains of the Rsp5 ubiquitin-protein ligase**. Mol Cell Biol 1999, **19**(1):342--352.

4. Kaida D, Toh-e A, Kikuchi Y: **Rsp5-Bul1/2 complex is necessary for the HSE-mediated gene expression in budding yeast**. Biochem Biophys Res Commun 2003, **306**(4):1037--1041.

5. Feller A, Boeckstaens M, Marini AM, Dubois E: **Transduction of the nitrogen signal activating Gln3-mediated transcription is independent of Npr1 kinase and Rsp5-Bul1/2 ubiquitin ligase in Saccharomyces cerevisiae**. J Biol Chem 2006, **281**(39):28546--28554.

6. Ren J, Kee Y, Huibregtse JM, Piper RC: **Hse1, a component of the yeast Hrs-STAM ubiquitin-sorting complex, associates with ubiquitin peptidases and a ligase to control sorting efficiency into multivesicular bodies**. Mol Biol Cell 2007, **18**(1):324--335.

7. Olsen JV, Blagoev B, Gnad F, Macek B, Kumar C, Mortensen P, Mann M: **Global, in vivo, and site-specific phosphorylation dynamics in signaling networks**. Cell 2006, **127**(3):635--648.

8. Tang L-Y, Deng N, Wang L-S, Dai J, Wang Z-L, Jiang X-S, Li S-J, Li L, Sheng Q-H, Wu D-Q et al: **Quantitative phosphoproteome profiling of Wnt3a-mediated signaling network: indicating the involvement of ribonucleoside-diphosphate reductase M2 subunit phosphorylation at residue serine 20 in canonical Wnt signal transduction**. Mol Cell Proteomics 2007, **6**(11):1952--1967.
